# A Comparative Study of Preloading With Ringer’s Lactate and Intravenous Ephedrine for the Prevention of Hypotension Due to Propofol During Induction of General Anesthesia

**DOI:** 10.7759/cureus.89097

**Published:** 2025-07-30

**Authors:** Megha B, Bhagyavardhan Botta, Kala Balasubramanian, Selvamani S, Kishore S

**Affiliations:** 1 Anesthesiology and Critical Care, Sree Balaji Medical College and Hospital, Chennai, IND

**Keywords:** ephedrine, general anesthesia, propofol, ringer’s lactate, spinal hypotension

## Abstract

Background

Propofol-induced hypotension during induction of general anesthesia remains a significant clinical concern, especially in healthy individuals. This study aimed to compare the effectiveness of two prophylactic interventions, crystalloid preloading with Ringer’s lactate and intravenous ephedrine, in maintaining hemodynamic stability during propofol induction in American Society of Anesthesiologists (ASA) I adult patients.

Methods

A prospective, interventional comparative study was conducted on 40 ASA I adult patients undergoing elective surgeries under general anesthesia. Patients were divided into two groups: Group A received Ringer’s lactate 10 mL/kg over 15 minutes prior to induction, while Group B received intravenous ephedrine 0.1 mg/kg one minute before induction. Hemodynamic parameters (heart rate (HR), systolic blood pressure (SBP), diastolic blood pressure (DBP), and mean arterial pressure (MAP)) were recorded at baseline, post-intubation, and at three, five, and seven minutes post-intubation.

Results

Demographic characteristics were comparable between groups. HR remained similar throughout, with no statistically significant differences. Group A showed significantly higher SBP at post-intubation (P = 0.0194) and at three minutes (P = 0.0051) than Group B. DBP was also significantly higher in Group A at three minutes (P = 0.0392) and five minutes (P = 0.0288). MAP values showed no significant differences at any time point, although Group B had slightly higher values at five and seven minutes.

Conclusion

Both interventions were effective in maintaining hemodynamic stability. However, Ringer’s lactate preloading showed more favorable SBP and DBP profiles in the early post-induction period, while ephedrine provided more consistent MAP trends without significant hypotension.

## Introduction

Propofol is a widely used intravenous anesthetic agent known for its rapid onset, smooth recovery profile, and antiemetic properties. These advantages have established it as a cornerstone drug in both inpatient and outpatient anesthesia practice. Despite its favorable pharmacokinetics, a significant limitation of propofol is its tendency to produce dose-dependent hypotension, even in physiologically normal patients undergoing elective procedures [1].

The hypotensive effect of propofol is primarily due to systemic vasodilation, myocardial depression, and attenuation of baroreceptor reflexes [2]. Hypotension after induction is due to impaired venous return as a result of positive pressure ventilation and positive end-expiratory pressure (PEEP). Mechanistically, it induces relaxation of vascular smooth muscle, leading to venous pooling, reduced preload, and consequently diminished cardiac output [3]. While these hemodynamic changes are often well tolerated, they may become clinically relevant during induction, especially when accompanied by airway manipulation or pre-existing hemodynamic instability.

To counteract these effects, several prophylactic strategies have been proposed. One conventional method is crystalloid preloading using isotonic solutions such as Ringer’s lactate, intended to augment intravascular volume and venous return. However, studies such as those by Turner et al. suggest that volume preloading alone may not adequately offset the vasodilatory and cardiac depressant effects of propofol [4].

An alternative approach involves the prophylactic administration of vasopressors. Ephedrine, a mixed alpha- and beta-adrenergic agonist, has garnered attention for its ability to increase systemic vascular resistance and enhance cardiac output. Ebert et al. demonstrated that propofol significantly blunts sympathetic tone, which may be restored by agents such as ephedrine [5]. Supporting this, Michelsen et al. observed that prophylactic ephedrine administration prior to induction significantly attenuated blood pressure drops, especially in elderly female patients [6].

Although both fluid preloading and vasopressor prophylaxis are commonly employed, there is limited comparative evidence on their effectiveness in low-risk surgical populations. El-Beheiry et al. highlighted that even brief episodes of hypotension during induction can negatively impact outcomes in emergency surgeries, stressing the need for reliable hemodynamic control [7]. While fluid preloading remains a simple and widely used strategy, its effects are transient and do not directly counteract the pharmacological mechanisms behind vasodilation. Moreover, fluid preloading also serves to correct fluid deficits resulting from preoperative fasting, which is routinely recommended to reduce the risk of pulmonary aspiration. Weeks emphasized that the decision between crystalloids and colloids for preloading should take into account factors such as cost, availability, and safety, considerations that are particularly important in resource-limited settings [8].

There is increasing consensus that pharmacological agents may provide more consistent hemodynamic stability. Both Ringer’s lactate and intravenous ephedrine are safe, low-cost, and widely available. As noted by Briggs et al., the hypotensive profile of agents such as propofol is not easily modifiable by preload alone, highlighting the need for vasopressor interventions [9]. Additionally, Chiu et al. found that while both ephedrine and metaraminol were effective in elderly patients, ephedrine provided superior cardiac output responses during induction [10].

This study is novel in directly comparing two mechanistically distinct interventions, crystalloid preloading versus intravenous ephedrine administration, in American Society of Anesthesiologists (ASA) I adult patients undergoing elective surgeries. By focusing on a low-risk cohort with standardized anesthetic protocols, the study aims to provide practical, evidence-based guidance for optimizing peri-induction hemodynamic stability in both resource-rich and resource-limited settings. This study aimed to evaluate the hemodynamic stability achieved with preloading using Ringer’s lactate prior to induction, assess the effectiveness of intravenous ephedrine in mitigating propofol-induced hypotension, and compare the changes in blood pressure and heart rate following both interventions during the induction and early maintenance phases of general anesthesia.

## Materials and methods

Study design and setting

This was a prospective, interventional, comparative study conducted in the department of anesthesiology at a tertiary care teaching hospital in Chennai, equipped with advanced perioperative monitoring and anesthesia facilities. The study was carried out over a period of six months, from October 2023 to March 2024, with the objective of comparing two prophylactic interventions for the prevention of hypotension during the induction of general anesthesia with propofol.

Ethical approval

Prior to initiation, the study protocol was reviewed and approved by the Institutional Human Ethics Committee (IHEC) of Sree Balaji Medical College and Hospital, in accordance with the ethical guidelines of the Indian Council of Medical Research (ICMR) (ethical clearance certificate number: 02211/SBMCH/IHEC/2022/2211). Written informed consent was obtained from all participants after providing a detailed explanation of the study objectives, procedures, potential risks, and benefits. Participants were assured that their data would be kept confidential and that they could withdraw from the study at any point without affecting their treatment.

Study population

The study included 40 adult patients of either sex, aged between 18 and 70 years, who were scheduled for elective surgeries under general anesthesia. All participants were classified as American Society of Anesthesiologists (ASA) I, indicating no significant systemic disease. These inclusion criteria were selected to ensure a homogenous population with minimal risk of baseline hemodynamic variability.

Patients were excluded if they had a history of cardiovascular, hepatic, renal, or endocrine disorders; were on antihypertensive therapy; or had known hypersensitivity to any of the study drugs. Patients undergoing emergency or high-risk surgeries were also excluded to eliminate confounding variables related to comorbid conditions or urgent interventions.

Sample size determination

A total of 40 patients were enrolled in the study. The sample size was determined based on findings from a prior study [11], which evaluated hemodynamic responses to prophylactic interventions during anesthetic induction, and a reduction in mean arterial pressure (MAP) > 20% from baseline was considered clinically significant. The study was powered to detect a clinically significant difference in mean arterial pressure (MAP) with a power of 80% and a significance level of 5%. Patients were sequentially enrolled and allocated into two comparative intervention groups based on the planned intraoperative management strategy.

Group allocation and interventions

The patients were divided into two groups, each consisting of 20 participants.

Group A (Crystalloid Group)

Patients received Ringer’s lactate at a dose of 10 mL/kg infused over 15 minutes prior to the induction of anesthesia. This intervention aimed to expand intravascular volume to counteract vasodilation induced by propofol.

Group B (Ephedrine Group)

Patients were administered intravenous ephedrine at a dose of 0.1 mg/kg as a single bolus injection one minute prior to the induction of anesthesia. Ephedrine, as a mixed alpha- and beta-adrenergic agonist, was used to enhance vascular tone and cardiac output.

These two interventions reflect commonly used clinical practices for mitigating hypotension during anesthetic induction. Group allocation was based on anesthesiologist discretion and clinical suitability, without randomization. No additional fluid boluses or vasopressors were administered during the first seven minutes following induction unless MAP fell below 60 mmHg, which was predefined as hypotension requiring rescue intervention.

Pre-anesthetic preparation

All patients underwent standard pre-anesthetic evaluation, including history taking, physical examination, and review of laboratory investigations. Standard fasting guidelines were followed prior to surgery. Upon arrival in the operating room, patients were connected to standard monitors including non-invasive blood pressure (NIBP), electrocardiogram (ECG), and pulse oximetry (SpO₂). Baseline vital parameters were recorded before any intervention was administered.

Anesthetic protocol

A uniform anesthetic protocol was followed for all patients to ensure consistency. All patients in the study were positioned supine with a slight head-up tilt during preloading and induction of anesthesia. No patients were placed in the lithotomy position at the time of blood pressure measurements. Premedication included intravenous midazolam 0.03 mg/kg for anxiolysis and fentanyl 2 µg/kg for analgesia. Anesthesia was induced with intravenous propofol 2 mg/kg, administered slowly over 30 seconds. Neuromuscular blockade was achieved with intravenous vecuronium 0.1 mg/kg to facilitate endotracheal intubation. Anesthesia was maintained with a 50:50 mixture of oxygen and nitrous oxide, along with isoflurane titrated to maintain a minimum alveolar concentration (MAC) appropriate for the surgical stimulus. Mechanical ventilation was initiated post-intubation and adjusted to maintain normocapnia.

Outcome measures

The primary outcome measure of the study was the change in mean arterial pressure (MAP) following the induction of anesthesia. Secondary outcomes included systolic blood pressure (SBP), diastolic blood pressure (DBP), and heart rate (HR). Hemodynamic variables were recorded at multiple time intervals to assess trends, including baseline (before induction), immediately after intubation, and at three, five, and seven minutes post-intubation. All measurements were obtained using a standardized multiparameter monitor to ensure consistency and reliability across the study population.

Statistical analysis

Data were compiled using Microsoft Excel (Microsoft Corp., Redmond, WA) and analyzed using IBM SPSS Statistics version 26.0 (IBM Corp., Armonk, NY). Continuous variables, such as MAP, SBP, DBP, and HR, were expressed as mean ± standard deviation (SD). Comparative analysis between the two groups was conducted using an unpaired Student’s t-test. A p-value of less than 0.05 was considered statistically significant. Graphical representations, including line charts, were used to visually demonstrate hemodynamic trends between the two intervention groups.

## Results

In Group A, six (30%) participants were aged 18-30 years, 10 (50%) were between 31 and 50 years, and four (20%) were in the 51-70 years range. Group B showed a similar distribution, with five (25%) in the 18-30 years, eight (40%) in the 31-50 years, and seven (35%) in the 51-70 years category. The mean age was 41.8 ± 15.28 years in Group A and 44.65 ± 17.57 years in Group B (P = 0.5873), showing no significant difference. Gender-wise, Group A had seven (35%) male participants and 13 (65%) female participants, while Group B included nine (45%) male participants and 11 (55%) female participants. These demographic characteristics presented in Table 1 were comparable, ensuring balanced baseline profiles and reducing potential bias in hemodynamic comparisons.

**Table 1:** Distribution of Age and Gender in the Study Population (N = 40)

Category	Subgroup	Group A (n = 20)	%	Group B (n = 20)	%
Age group (years)	18-30	6	30%	5	25%
	31-50	10	50%	8	40%
	51-70	4	20%	7	35%
Gender	Male	7	35%	9	45%
	Female	13	65%	11	55%

At baseline, the mean HR was 83.5 ± 21.7 bpm in Group A and 81.1 ± 18.0 bpm in Group B (P = 0.7056). Post-intubation, HR rose slightly to 86.8 ± 16.7 bpm in Group A and 88.8 ± 16.7 bpm in Group B (P = 0.8006). At three minutes, values were 84.6 ± 13.9 bpm and 83.2 ± 13.2 bpm, respectively. At five minutes, Group A had 82.1 ± 12.2 bpm and Group B 83.6 ± 13.6 bpm. At seven minutes, HR dropped to 78.6 ± 11.9 bpm in Group A and remained at 82.3 ± 15.8 bpm in Group B. None of these differences were statistically significant (all P > 0.4). This suggests that both interventions maintained hemodynamic chronotropy without inducing reflex tachycardia or bradycardia (Table 2).

**Table 2:** Comparison of Mean HR at Different Time Intervals Between Group A and Group B (N = 40) Student t-test p < 0.05: significant HR: heart rate, SD: standard deviation

Time interval	Group A (mean ± SD)	Group B (mean ± SD)	p-value
Before induction	83.5 ± 21.7	81.1 ± 18.0	0.7056
After intubation	86.8 ± 16.7	88.8 ± 16.7	0.8006
3 minutes	84.6 ± 13.9	83.2 ± 13.2	0.7457
5 minutes	82.1 ± 12.2	83.6 ± 13.6	0.7155
7 minutes	78.6 ± 11.9	82.3 ± 15.8	0.4081

Baseline SBP was slightly higher in Group A (138.6 ± 27.9 mmHg) than in Group B (127.8 ± 18.4 mmHg), although not statistically significant (P = 0.1486). Post-intubation SBP was significantly higher in Group A (140.7 ± 25.6 mmHg) versus Group B (127.5 ± 24.1 mmHg) (P = 0.0194). At three minutes, the difference remained significant: 135.5 ± 22.8 mmHg (Group A) versus 117.9 ± 13.4 mmHg (Group B) (P = 0.0051). No significant differences were seen at five minutes (128.4 ± 23.2 versus 120.5 ± 17.7 mmHg) or seven minutes (123.9 ± 22.8 versus 117.8 ± 15.5 mmHg). These findings indicate that ephedrine effectively mitigated the typical SBP drop seen with propofol (Table 3).

**Table 3:** Comparison of Mean SBP at Different Time Points Between Group A and Group B (N = 40) Student t-test p < 0.05: significant SBP: systolic blood pressure, SD: standard deviation

Time point	Group A (mean ± SD)	Group B (mean ± SD)	p-value
Before induction	138.6 ± 27.9	127.8 ± 18.4	0.1486
After intubation	140.7 ± 25.6	127.5 ± 24.1	0.0194
3 minutes	135.5 ± 22.8	117.9 ± 13.4	0.0051
5 minutes	128.4 ± 23.2	120.5 ± 17.7	0.2335
7 minutes	123.9 ± 22.8	117.8 ± 15.5	0.3277

Baseline DBP was similar between groups: 76.1 ± 11.5 mmHg (Group A) and 73.8 ± 12.7 mmHg (Group B) (P = 0.5518). Post-intubation DBP dropped to 78.0 ± 12.3 mmHg and 71.2 ± 11.3 mmHg, respectively (P = 0.0765). At three minutes, Group A recorded 75.8 ± 10.7 mmHg, whereas Group B maintained 72.9 ± 10.4 mmHg (P = 0.0392). A significant difference persisted at five minutes: 76.1 ± 11.1 mmHg (Group A) versus 71.8 ± 14.0 mmHg (Group B) (P = 0.0288). These results show ephedrine’s superior efficacy in maintaining diastolic tone during anesthetic induction (Table 4).

**Table 4:** Comparison of Mean DBP at Different Time Points Between Group A and Group B (N = 40) Student t-test p < 0.05: significant DBP: diastolic blood pressure, SD: standard deviation

Time point	Group A (mean ± SD)	Group B (mean ± SD)	p-value
Before induction	76.1 ± 11.5	73.8 ± 12.7	0.5518
After intubation	78.0 ± 12.3	71.2 ± 11.3	0.0765
3 minutes	75.8 ± 10.7	72.9 ± 10.4	0.0392
5 minutes	76.1 ± 11.1	71.8 ± 14.0	0.0288
7 minutes	71.5 ± 9.8	73.5 ± 10.3	0.0533

MAP at baseline was similar: 96.05 ± 18.3 mmHg (Group A) and 95.5 ± 13.0 mmHg (Group B) (P = 0.8509). Following intubation, values were 91.0 ± 13.2 and 91.4 ± 7.36 mmHg (P = 0.9064), and at three minutes, 85.3 ± 10.8 (Group A) versus 86.7 ± 7.8 mmHg (Group B) (P = 0.6411). At five and seven minutes, Group B consistently showed slightly higher MAPs (94.2 ± 16.8 and 91.4 ± 15.6 mmHg) than Group A (89.9 ± 16.8 and 91.5 ± 19.9 mmHg), although not statistically significant. These stable MAP trends suggest that both interventions were effective, with ephedrine offering a marginal advantage in maintaining perfusion pressure (Table 5).

**Table 5:** Comparison of MAP at Different Time Points Between Group A and Group B (N = 40) Student t-test p < 0.05: significant MAP: mean arterial pressure, SD: standard deviation

Time point	Group A (mean ± SD)	Group B (mean ± SD)	p-value
Before induction	96.05 ± 18.3	95.5 ± 13.0	0.8509
After intubation	91.0 ± 13.2	91.4 ± 7.36	0.9064
3 minutes	85.3 ± 10.8	86.7 ± 7.8	0.6411
5 minutes	89.9 ± 16.8	94.2 ± 16.8	0.4233
7 minutes	91.5 ± 19.9	91.4 ± 15.6	0.9860

**Figure 1:**
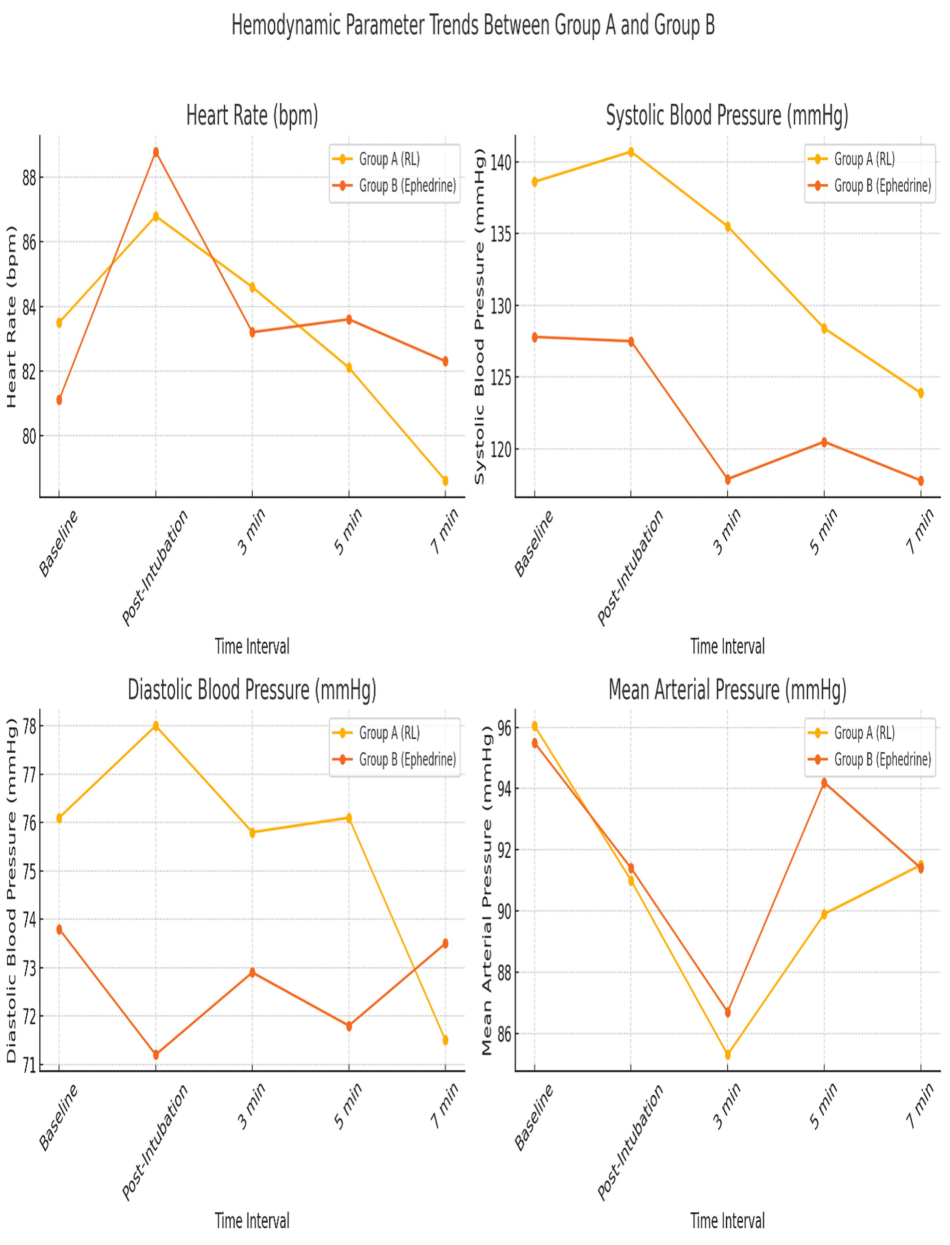
Line Graph Showing Comparison of Heart Rate, Systolic Blood Pressure, Diastolic Blood Pressure, and Mean Arterial Pressure Between the Two Groups at Various Time Intervals

Figure 1 presents a line diagram comparing heart rate, mean SBP, DBP, and mean arterial pressure between the two groups. The graph illustrates hemodynamic trends over time. Variations at different time points are clearly observable.

## Discussion

This study evaluated and compared the effectiveness of two commonly used prophylactic interventions, crystalloid preloading with Ringer’s lactate and intravenous ephedrine, in preventing hypotension following induction with propofol in ASA I adult patients undergoing elective surgeries. By assessing hemodynamic parameters at multiple time points, the study aimed to determine which approach offered better peri-induction cardiovascular stability in a low-risk surgical population.

Propofol is a commonly used induction agent favored for its rapid onset and favorable recovery profile. However, its most recognized drawback is dose-dependent hypotension resulting from both myocardial depression and systemic vasodilation. Smith et al. highlighted that the cardiovascular depressive effect of propofol, although well-tolerated in healthy individuals, poses a significant risk in susceptible patients, especially during the induction phase when compensatory mechanisms may be blunted [1]. The importance of mitigating this hypotensive response is underscored by Hug et al., who demonstrated through analysis of over 25,000 patients that MAP reductions during induction are both common and clinically significant [2].

In the present study, heart rate was not significantly different between the Ringer’s lactate and ephedrine groups at any measured time point. This is in agreement with the findings of Michelsen et al., who reported that ephedrine use preserved hemodynamic stability without inducing reflex tachycardia, especially in elderly female patients [6]. Similarly, El-Beheiry et al. demonstrated that prophylactic administration of vasopressors did not significantly alter heart rate, suggesting a favorable chronotropic profile when used judiciously [7]. This preservation of heart rate suggests that neither intervention led to adverse sympathetic overactivation or pharmacological bradycardia, which is crucial in maintaining myocardial oxygen demand during induction.

Our study found a statistically significant preservation of SBP in the ephedrine group at two key time points, immediately post-intubation and at three minutes. These results are congruent with the outcomes reported by Chiu et al., who found that prophylactic metaraminol and ephedrine were effective in preventing sharp SBP drops in elderly surgical patients, with ephedrine offering a better cardiac output profile [10]. The findings of Kasaba et al. also support our results, as they demonstrated that ephedrine consistently maintained SBP better than dopamine or dobutamine during anesthesia involving neuraxial blockade and concurrent propofol administration [11]. By contrast, Group A (Ringer’s lactate) experienced a transient dip in SBP, which aligns with Turner et al., who observed that crystalloids alone fail to adequately counteract the vasodilatory effects of propofol in certain cohorts [4].

Although MAP differences were not statistically significant at any time point in our study, Group B (ephedrine) maintained consistently higher MAP values. These findings mirror those of Coates et al., who documented that MAP is best preserved when fluid loading is combined with propofol titration or pharmacological agents [12]. Interestingly, Claeys et al. reported that MAP fluctuations with propofol could be blunted by multimodal interventions, suggesting a ceiling effect with fluid loading alone [13]. Our findings support the notion that although Ringer’s lactate offers transient MAP buffering, ephedrine provides more consistent vascular tone maintenance.

Studies such as those by Monk et al. [14] and Stephan et al. [15] have emphasized the patient-specific variability in hemodynamic responses to propofol. Monk et al. noted that patients with lower autonomic reserve, such as the elderly or those with subclinical cardiac dysfunction, experience exaggerated hypotensive responses despite preloading [14]. Stephan et al. demonstrated that propofol-induced hypotension was significantly more pronounced in patients with coronary artery disease, raising concerns for intraoperative ischemia [15]. While our study population included ASA I patients, these insights are clinically relevant and reinforce the generalizability of ephedrine as a robust prophylactic agent, particularly as patient comorbidities increase.

Ephedrine’s ability to preserve DBP significantly at three and five minutes post-induction in our study aligns with previous observations regarding its potent vasoconstrictive properties. Michelsen et al. illustrated the benefit of ephedrine in counteracting propofol-induced vasodilation, which was especially evident in preserving diastolic pressure [6]. Robinson et al. described how propofol induces vasodilation by direct vascular smooth muscle relaxation and sympathoinhibition, mechanisms not fully countered by fluids alone [16]. The superior DBP values seen in our ephedrine group may thus reflect its action at both α-adrenergic receptors and on cardiac output.

Our findings support the conclusion that crystalloid preloading alone may be insufficient to prevent hypotension in the early post-induction period. Al-Ghamdi showed that even hydroxyethyl starch, a colloid with theoretically better volume-expanding capacity, failed to prevent hypotension during induction with propofol and fentanyl [17]. This underlines the pharmacological nature of propofol’s cardiovascular effects, which cannot be adequately countered by volume status manipulation alone. Furthermore, Weeks questioned the role of colloids versus crystalloids, emphasizing that while colloids may offer transient benefits, they do not prevent propofol-induced hypotension unless combined with vasopressors [8].

Future research should include larger, multicentric trials with diverse ASA categories, particularly involving elderly and comorbid patients. Studies assessing long-term intraoperative and postoperative hemodynamic outcomes are warranted. Comparative studies incorporating combined interventions (fluid preloading plus vasopressors) may offer a more robust prophylactic approach. Additionally, evaluation of cost-effectiveness and safety profiles in broader populations would enhance clinical applicability.

This study had a few limitations. First, it was conducted in a single tertiary care center, which may limit the generalizability of the findings. Second, the study population included only ASA I patients undergoing elective surgeries, excluding those with comorbidities or high-risk conditions where hemodynamic changes may be more pronounced. Third, as a non-randomized study, there is potential for selection bias in group allocation. We did not standardize or control for airway pressure and positive end-expiratory pressure (PEEP) levels during the study. This may have introduced variability in hemodynamic measurements, particularly post-intubation.

## Conclusions

This study demonstrated that prophylactic administration of intravenous ephedrine is more effective than crystalloid preloading with Ringer’s lactate in attenuating hypotension following the induction of general anesthesia with propofol in ASA I patients. Ephedrine significantly preserved both systolic and diastolic blood pressures during the critical early post-induction period, without inducing adverse changes in heart rate. While both interventions maintained mean arterial pressure within acceptable clinical ranges, ephedrine offered superior hemodynamic stability. These findings support the preferential use of ephedrine in routine anesthetic practice to enhance cardiovascular safety during induction, particularly in settings where resource efficiency and patient stability are paramount.
